# Repetition leads to short-term reduction of word frequency and name agreement effects: Evidence from a Dutch two-session picture naming experiment

**DOI:** 10.1177/17470218251365517

**Published:** 2025-08-02

**Authors:** Caitlin Decuyper, Ruth E Corps, Antje S Meyer

**Affiliations:** 1 Max Planck Institute for Psycholinguistics, Nijmegen, the Netherlands; 2 University of Sheffield, Sheffield, UK; 3 Donders Institute for Brain, Cognition, and Behaviour, Radboud University, Nijmegen, the Netherlands

**Keywords:** Word production, word frequency, name agreement, repetition priming, lexical access

## Abstract

Word frequency (WF) and name agreement (NA) affect a word’s accessibility during speech production. Speakers are faster to name pictures with high-frequency (e.g. *dog*) compared to low-frequency names (e.g. *rhinoceros*) and those that a group of speakers tend to agree on the name of (high NA; e.g. *arm*) than those that they do not (low NA; e.g. *sofa, couch*). Recent accounts of lexical access suggest that the structure of the mental lexicon is flexible and changes with exposure. Consistent with this view, repetition priming studies have shown that low-frequency and low NA items benefit from repetition more than high-frequency and high NA items. But there is little evidence that repetition has long-term effects on WF and NA. We tested this issue in a two-session (online) picture naming study. In Session 1, participants named pictures varying in WF and NA three times each, and so we could test the short-term effects of repetition on WF and NA. We tested long-term effects of repetition by having participants name the same old items 1 week later in Session 2, together with new items that they had not named previously. In Session 1 the WF effect was eliminated by repetition, while the NA effect was reduced but still present. Thus, previous naming affected both the WF and NA effects. However, both effects reappeared in Session 2. These findings suggest that previous naming can reduce the WF and NA effect, thus affecting how easy it is to produce a word, but these effects are relatively short-lived.

## Introduction

When a speaker names an object, they initiate several processes involved in speech planning. First, they perceptually process the object they want to name (e.g. identifying a cupcake). Then they create conceptual links to the object (e.g. edible, cake-like, sweet) in order to select the object’s conceptual representation and retrieve its corresponding lexical representation (e.g. *cupcake*, not *muffin*). Many contemporary models of lexical access assume that the latter step involves a cascade of distinct processes, leading to the retrieval of grammatical, morphological and phonological information about the word. Finally, articulatory planning processes are necessary to produce the selected word with the correct pronunciation (e.g. ‘kʌpkeɪk’; Dell et al., 1997; Goldrick & Rapp, 2002; Levelt et al., 1999; Oppenheim et al., 2010). There are several variables that can affect these processes and the time they take to complete, typically measured using response times. This study focuses on two variables that are known to affect the ease of word retrieval: word frequency, which refers to the frequency of an object’s name, and name agreement, which refers to the degree to which a group of speakers agrees on the name of a particular object.

Word frequency (WF) is a corpus-based lexical variable that refers to how often a word occurs in corpora of text, such as books, newspapers or subtitles (Brysbaert et al., 2018). These corpus frequencies are thought to correspond to how often speakers encounter words in daily life. Previous research has shown that speakers are faster to name pictures with more frequent names (e.g. *dog*) compared to items with less frequent names (e.g. *rhinoceros*; e.g. Alario et al., 2004; Oldfield & Wingfield, 1965). This WF effect is very robust and has been replicated many times, and is thought to occur because words that are encountered more frequently are easier to access and produce. Some theories of lexical access (e.g. Levelt et al., 1999) ascribe this difference in accessibility between low and high-frequency words to a difference in their baseline activation. High-frequency items have a higher baseline activation, and therefore reach the activation threshold for production faster, which makes them easier to produce. In other models, frequency differences are captured in the connection strengths between conceptual and lexical units, with high-frequency words having stronger connections to conceptual representations than low-frequency words (e.g. Dell et al., 1999).

Another strong predictor of naming latency is name agreement (NA). This value is typically a percentage, which represents the proportion of speakers within a sample that agree on a particular name for an object. NA is higher when participants tend to provide the same object name (e.g. most speakers refer to a picture of an arm as *arm*), but lower when they tend to differ in names (e.g. both *sofa* and *couch* could be an accurate name for the same picture). Research suggests that speakers are faster to name pictures with high NA compared to pictures with low NA (e.g. Alario et al., 2004; Vitkovitch & Tyrrell, 1995). This NA effect is thought to occur because high and low NA items have a different number of competing conceptual and/or lexical representations (Levelt et al., 1999). Pictures with low NA strongly activate multiple lexical concepts and the corresponding names. A related assumption is that speakers not only consider their preferred name for an object (individual-level NA) but are also aware of how other speakers in their community would name this object (population-level NA), creating a different type of competition between names (see Balatsou et al., 2022). In both views, selecting one name out of several alternatives takes time and, as a result, slows down naming.

### WF and NA altered by experience

A word’s WF and NA affect its accessibility. Both WF and NA are typically defined using corpora. It is assumed that counts of frequency or name preferences from a wider population represent WF and NA within an individual speaker’s mind. However, representations of these variables are likely to change with the individual’s experience with language. In particular, low-frequency words can become more frequent with use (the frequency ‘counter’ goes up, e.g. Levelt et al, 1999). Repeated naming can increase a word’s baseline activation, or the strength between the relevant conceptual and lexical representations, which makes it easier to produce next time. Repeatedly using the same name for items with low NA can create a bias towards a preferred name, and so alternatives might be considered to a lesser extent, which potentially reduces competition between them. Some picture-word interference studies (see, for instance, Miozzo & Caramazza, 2003; Wöhner et al., 2024) have shown that repetition can reduce interference from related distractor words. For instance, in a study by Wöhner et al. (2024, Experiment 2), participants were slower to name pictures (e.g. *duck*) accompanied by semantically related distractor words (e.g. *eagle*) compared to unrelated distractors. This difference in response times (i.e. the semantic interference effect) was reduced with repeated naming of the targets. These findings suggest that non-selected semantically related competitors can become less available with repetition.

Evidence that picture naming benefits from repetition comes from repetition priming studies. Repetition results in faster response times when an item is named a second time (see e.g. Francis, 2014, for a review). The studies summarised below suggest that this repetition benefit can also modulate the WF and NA effect. So far, it remains unclear whether repetition has any *long-lasting* effects on the WF and NA effect that could alter the long-term structure of the mental lexicon.

### WF and repetition priming

Jescheniak and Levelt’s (1994) seminal study of the WF effect reported stable WF effects across three repetitions of the same items in a picture naming task. This pattern was replicated in two studies that used the same materials. One study (Levelt et al., 1998, pilot picture naming experiment outside of MEG scanner) found stable WF effects even after 12 repetitions. A stable WF effect was also reported when participants named the same pictures again 6 months later. Meyer et al. (1998) found a similar WF effect across eight presentations. Another picture naming task in a study by Paucke et al. (2015) resulted in a stable WF effect across four repetitions.

However, other picture naming studies, including a replication of Jescheniak and Levelt’s study by Corps and Meyer (2023), did report an interaction between the WF effect and repetition, indicating the WF effect can be reduced with repeated presentation of the same items. In particular, Corps and Meyer (2023) found that the WF effect was larger on an item’s first presentation compared to when participants named the picture for the second or third time. Similarly, Griffin and Bock (1998) found a WF effect on the first and second presentation of low and high frequency items, but not on the third presentation. A study by La Heij et al. (1999) reported a larger WF effect in the first block (first and second presentation) compared to the second block (third and fourth presentation) of two experiments. Thus, there is some evidence that the WF effect during naming can be reduced with repetition of the same pictures.

### NA and repetition priming

Regarding NA, mixed results are reported as well. Some studies have found a reduction of the effect after the first presentation (e.g. Park & Gabrieli, 1995), whereas others, for instance, Alario et al. (2004), found similar NA effects on each of two repetitions of an item. A study by Corps and Meyer (2025a), which tested the interaction between repetition and NA more systematically found that repeated naming reduced but did not eliminate the NA effect. Participants were asked to name the same pictures three times each after a familiarisation phase. Participants were faster to name high NA items than low NA items on all three repetitions. The difference in response times for high and low NA items decreased with repetition, as low NA items benefited more from repetition than high NA items. However, the NA effect could not be eliminated. In sum, repetition generally benefits word production, and there is some evidence that it can modulate WF and NA effects.

### Long-term effects of repetition

Findings from the literature on repetition priming are consistent with recent dynamic theories of lexical access that assume that the structure of the mental lexicon is flexible and can change with experience (e.g. Damian & Als, 2005; Howard et al., 2006; Oppenheim et al., 2010). Every time an object is named, the link between the depicted concept and the selected word is strengthened, and the links with activated but unselected words are weakened. Thus, with every encounter of a word, a trace is left in memory. If these traces are durable, a word’s representation can become more accessible over time, which explains why frequent words can be produced faster than less frequent ones, why low NA slows down naming (there are multiple competing names for an object), and why the modal names of objects with low NA are produced faster than alternate names. However, little is known about *long-term* effects of repetition on a word’s accessibility. It is unclear whether memory traces created by repeated naming are durable enough to support cumulative learning and therefore contribute to the emergence of WF and NA effects. Testing this claim is important because it provides insight into the way the structure of the lexicon changes with experience.

To the best of our knowledge, there are no studies that have directly investigated long-term effects of repetition on the NA effect, and only a few studies have considered the long-term effects of repetition for the WF effect. In a study by Tsuboi et al. (2021), participants were asked to read picture names with varying word frequency during an encoding phase and later name the corresponding pictures after a retention interval of either 10 min (within Session 1) or 1 week (Session 2). In Session 1, they found a repetition priming effect that was stronger for low-frequency items compared to high-frequency items. This repetition priming effect was no longer seen in Session 2. Participants were only exposed to each picture name once in Session 1, and so this might not have been enough to lead to more long-term repetition priming effects that could have affected response times in Session 2.

One two-session study by Corps and Meyer (2025b) looked at the effect of repetition on the WF effect within and across experimental sessions. In their Experiment 1, participants first named pictures with high- and low-frequency names six times each. In a second and third session, about a week apart, they named the pictures once more, along with new pictures. There was a WF effect in the first two presentations of the pictures in Session 1, but not in the following presentations. Importantly, in Sessions 2 and 3, a frequency effect was only seen for the new items, but not for those that had been presented in Session 1. In Experiment 2, participants first read the picture names and carried out a semantic judgement task, again six times for each item. In Sessions 2 and 3 they named pictures, as in Experiment 1. Again, a WF effect on picture naming was only found for the new items, but not for the old items, whose names had been read in Session 1. These results indicate that using the picture names repeatedly can create a memory trace that is durable enough to support longer-term lexical learning.

### Comparing WF and NA

Results from repetition priming studies show that the WF effect as well as the NA effect can be reduced by repetition and that repetition can have relatively long-lasting effects on a word’s accessibility. However, the WF effect seems to be easier to overrule, as it can disappear after only a couple of repetitions, and can still be absent days later, whereas the more robust NA effect can be reduced but not eliminated even after multiple repetitions. This pattern hints at different learning mechanisms behind WF and NA effects and requires further research. In particular, memory traces created by repeated naming might be durable enough to support cumulative learning for an individual’s representation of an item’s WF but not NA.

One possible explanation for the difference in effects of repetition is that the representation of WF in memory is mostly based on an individual’s experience with language, that is, the individual’s frequency counter is updated with every instance of a word, whereas stored information about NA is partly based on population-level knowledge. Even though individual speakers might have a preferred name to refer to an object, knowledge about how others might name the object is thought to be represented in the speakers’ minds as well. This idea was tested in a study by Balatsou et al. (2022) where speakers named high and low NA items twice. They used more modal names (i.e. names preferred by participants in a prior norming study) than alternative names on both repetitions and tended to use the same name on both occasions. If they switched names for high NA items, it was more likely that they switched to a name that was more commonly used. Furthermore, response time analyses in the study by Corps and Meyer (2025a) showed faster responses for modal compared to alternative names for pictures with low NA, providing further evidence for some level of representation of community-level NA. These findings suggest that population-level NA is also represented, and speakers do not only activate their preferred name but also consider the most common name for an item within their community. This competition between multiple plausible names slows down production for low NA items. Population-level knowledge about NA is likely to be difficult to override with repetition, which might be why low NA items benefit less from repeated naming compared to low WF items.

### Current study

The current study builds on earlier work by Corps and Meyer (2025a, 2025b) and aims to directly test short- and long-term effects of repetition on the WF and NA effect within the same picture naming task. Experiment 1 was an Object Recognition Task that included the pictures we intended to use in the naming task. We wanted to assess whether the WF and NA of an object’s name predicts how fast this object is recognised and exclude any items that were particularly difficult to recognise. On each trial of the experiment, participants first saw a word and then a picture and, upon picture presentation, had to indicate as quickly as possible whether the word was the name of the object. Stadthagen-Gonzalez et al. (2009) used the same task and examined the effects of a large set of visual, conceptual and lexical variables on object recognition times for line drawings. They did not find any effect of WF or NA, and we did not expect to find such effects either.

In Experiment 2, a large group of participants named pictures with varying WF and NA (both treated as continuous variables) in a two-session online experiment. Session 2 was administered 5 to 7 days after Session 1. In Session 1, pictures were named three times each to look at within session effects of repetition. The same (*old*) pictures were named again in Session 2, interleaved with *new* items.

This within-participant design that included both variables allows us to assess the attenuation of WF and NA effects through repetition in the same experimental context. An individual’s representations of both variables have the potential to change with experience. However, few studies have directly tested the effect of repetition on WF and NA effects. Furthermore, results from previous research suggest that the WF effect is more susceptible to repetition than the NA effect. This study aims to further explore this dissociation of effects.

The comparison of results for WF and NA could also inform us about potentially different learning mechanisms behind stored information about a word’s WF and NA. If recent naming affects a word’s accessibility, we expect both the WF and NA effects to attenuate with repetition, as predicted by cumulative learning. If repetition creates memory traces that are durable enough to alter the representation of a word’s WF or NA long-term, the effect should still be reduced for *old* items in Session 2. However, cumulative learning through experience might affect WF more than NA if, for instance, community knowledge about a word’s NA is harder to overrule than a word’s frequency ‘counter’.

This study will also make a methodological contribution. We used the Bank of Standardised Stimuli (BOSS) database (Decuyper et al., 2021) to select stimuli for this study. NA percentages included in this database were based on written responses. We will validate these percentages by adding and comparing NA based on spoken responses of a larger subset of speakers. Furthermore, we have added naming latencies for verbal responses and object recognition times collected in this study to a subset of items in the database.

## Experiment 1: object recognition

In Experiment 1, we used an object recognition task (also referred to as word-picture or name-picture verification or matching task) to assess whether the WF and NA of an object’s name predicts how fast this object would be recognised and to check whether any items would be particularly difficult to recognise. In an online experiment, participants first saw a word and immediately afterwards a picture and then indicated via button press whether or not the picture matched the word. This task was designed to test perceptual and conceptual processing.

### Methods

#### Participants

Thirty-one native speakers of Dutch (9 females, 22 males; *M* age = 26 years) took part in this online study through Prolific Academic. They were rewarded £3.50 for their participation. All participants had a minimum 90% satisfactory rate from prior assignments in prolific. Participants reported no language-related disorders. Ethical approval for the study was given by the Ethics Board of the Social Sciences Faculty at Radboud University. We discarded data from 2 participants because they withdrew from the experiment before finishing all trials, and so we analysed data from 29 participants.

#### Materials

For the experimental trials, in which the word matched the picture, we selected 246 pictures and their descriptive statistics from the Dutch BOSS (Decuyper et al., 2021), which is a database of coloured photographs of everyday objects (see Table A1 of the Supplemental Material for a full list of items). To ensure we used pictures that are easy to recognise, we only selected items with a DKO-score (Do not Know the Object) of 0, which means all participants in the BOSS norming study (*n* = 50–52 per items) had recognised the item, and a DKN (Do not Know the Name) score of less than 5%, which means that less than 5% of the participants did recognise the depicted object, but could not think of the object’s name. In addition, all items had an average Object Agreement score of 4 or higher. Object Agreement is a five-point rating of how well participants thought the picture represented its concept. Items varied in NA and WF. NA refers to the percentage of participants who agree on the dominant name for a picture and was retrieved from the BOSS database (Decuyper et al., 2021). WF is measured in Zipf scores, which is a logarithmic scale ranging from 1 (*extremely low frequency*) to 7 (*very high frequency*). Zipf is derived from SUBTLEX(-NL) frequency (see van Heuven et al., 2014) and aggregated across parts of speech. WF (*M* Zipf = 3.38, *SD* = 0.86, range = 1.66–4.98) and NA (*M* NA = 74.55, *SD* = 19.51, range = 24–100) were defined as two continuous variables. WF and NA were not correlated (*r* = .04, *p* = .51).

In addition, we selected 246 pictures that served as filler items in the experiment. Fillers always prompted a *no* response. We used names of other filler items to create a mismatch between the word and the picture. The paired word and the picture name were semantically and phonologically unrelated. Filler items had a range of values for WF (*M* Zipf = 3.27, *SD* = 0.73, range = 1.66–5.49) and NA (*M* NA = 75.87, *SD* = 24.18, range = 22–100) as well.

#### Design

The 492 items (246 experimental items requiring a *yes* response and 246 filler items requiring a *no* response) were pseudorandomised using Mix (van Casteren & Davis, 2006) and split into two lists. Participants were randomly assigned to one list. Each item was presented once and was never immediately preceded by the presentation of a phonologically, semantically or associatively related item. No more than three items of the same type (experimental or filler trial) were presented in adjacent trials.

#### Procedure

Data were collected online using Frinex (FRamework for INteractive EXperiments, a software package developed for running experiments by the technical group at the Max Planck Institute for Psycholinguistics in Nijmegen). Participants were encouraged to complete the experiment in a quiet environment, away from any distractions such as their phone or television. Each trial began with a fixation cross (+) presented in the centre of the screen for 500 ms. The word appeared 300 ms later and stayed on-screen for 2,000 ms. Then, the word was replaced with the target picture. Participants responded *yes* (M key on their keyboard, ‘Horen wel bij elkaar’) if the word and the picture matched (target trials), or *no* (Z key on their keyboard, ‘Horen niet bij elkaar’) if they did not match (filler trials). They had 2,000 ms to respond. The next trial began 1,500 ms later, either after the participant had responded or after the timeout. The experiment started with four practice trials to familiarise themselves with the task. The main experiment consisted of 2 blocks of 123 trials. Participants could take a break between blocks.

### Data analysis

Participants’ response times (RTs) were measured from picture onset. We discarded incorrect responses (*n* = 210; 3%) and trials with no response (*n* = 71; 1%) prior to analysis. All participants responded well above chance level (M = 96%). The remainder of the analysis only included button press times for experimental trials (yes responses, *n* = 3417). We discarded RTs for 35 target pictures before analysis because these were pre-tested for another experiment. Finally, we removed 2.61% of remaining responses as outliers (77 responses, which were slower than 2.5 *SD*s above the participant’s mean and two extremely fast responses that were less than 20 ms and thus unlikely to be meaningful responses).

Effects of WF and NA on button press RTs were tested using the *lmer* function of the *lme4* package (version 1.1-33, Bates et al., 2015; RStudio version 4.3.0, R Core Team, 2020). We selected the maximal converging model (Barr et al., 2013). Random effects were maximal without overparameterisation. In particular, we did not accept models throwing singular fit warnings and avoided high correlations between random effects. Raw data, analysis script, and full model structure and output are available at https://osf.io/b87qz/.

### Results and discussion

**Figure 1.**
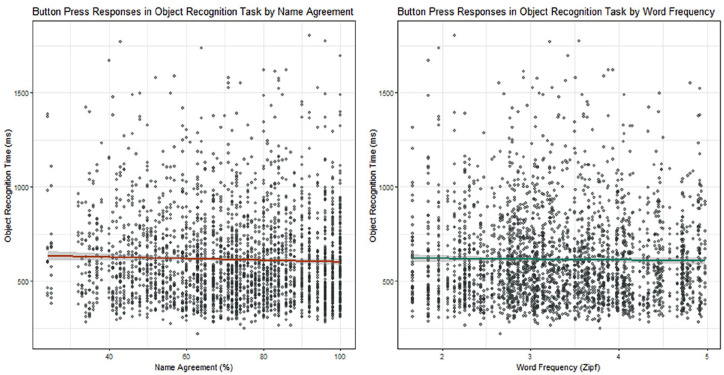
Distribution of object recognition times by name agreement (left) and word frequency (right).

On average, participants responded 614 ms (*SD* = 243) after picture onset. This object recognition task (E1) was used as a pre-test for the materials in our online naming study (E2) and therefore included more items than we intended to use in Experiment 2. After calculating mean RTs per picture, we discarded the five items with the slowest mean RT to ensure pictures in E2 would be easy to recognise. We excluded 10 additional items to adjust the continuum of NA scores, which was slightly skewed towards high NA in E1. Reported statistical test and graphs in the remainder of the analysis of E1 thus include data for the 196 pictures we will use in E2 (2,678 data points). Mean RT for the remaining 196 items was 614 ms (*SD* = 241). We fitted a model that included main effects of modal NA and Zipf WF and the interaction between these two variables.^1^ The random effects structure consisted of intercepts for subject and picture. The model yielded no main effects (NA: β = −.90, *t* = −0.96, *p* = .34; WF: β = −11.26, *t* = −0.59, *p* = .56) and no interaction between NA and WF (β = .14, *t* = 0.53, *p* = .60); Pearson correlation tests showed no correlation between reaction time and NA (*r* = −.03, *t* = −1.74, *p* = .08) or reaction time and WF (*r* = −.01, *t* = −0.72, *p* = .47), indicating that pictures with a higher name agreement or a more frequent name were not recognised faster than pictures with lower NA or frequency (see Figure 1). In sum, for this set of pictures, recognition times were not modulated by the frequency of the picture name or the degree of name agreement (consistent with Stadthagen-Gonzalez et al., 2009), and so possible effects of WF and NA on naming latencies in Experiment 2 will not be confounded by differences in perceptual processing.

## Experiment 2: two-session online picture naming

In Experiment 2, we used a two-session picture naming task to assess whether repetition priming affects WF and NA effects to a similar extent (Session 1) and to study the stability and longevity of repetition-related changes in WF and NA (Session 2; 5–7 days after Session 1). Participants were not familiarised with the pictures and so, for low NA pictures, were free to select their preferred name. In the first analysis, we included the response latencies for all appropriate names and related them to the corresponding lexical frequencies and indicators of NA. The latter variable was based on the responses of the current sample rather than the norms used to select the stimuli (see below for details). As the participants named the items several times, we also examined whether they consistently used the same name or switched names, and in a second analysis, only included the sets of trials where participants had consistently used the same name to refer to an object. For such trials, one might expect particularly pronounced repetition effects and therefore strong modifications of the NA and WF effect. Finally, we compared RTs for modal names (those preferred in the sample) and correct alternative names to test whether speakers consider knowledge about an item’s modal name during picture name (see Balatsou et al., 2022).

### Methods

#### Participants

Data collection continued until 98 participants had taken part in both Session 1 and Session 2, and audio recordings for both sessions were of sufficient quality to define response onsets. Participants were native speakers of Dutch (60 female, 37 male, 1 non-binary, *M* age = 28 years) and were recruited online using Prolific Academic (*n* = 71) and the Max Planck Institute participant pool (*n* = 27). They received £8.5 (Prolific) or €10 (MPI) for participating in both sessions. Those who were recruited through Prolific all lived in the Netherlands and had a minimum 90% ‘satisfactory’ rate of performance based on prior experiments. Participants reported no speech impairments. Ethical approval for the study was given by the Ethical Board of the Social Sciences Faculty at Radboud University.

#### Materials

Half of the 196 pictures that we selected after the Object Recognition task (E1) were used for Session 1, while the other half were introduced in Session 2 (*new* items). The pictures presented during Session 1 served as *old* items in Session 2. Old and new items were matched for NA (*t*(97) = 0.22, *p* = .82), WF (*t*(97) = −0.22, *p* = .83), mean Object Recognition time (collected in E1, OR RT, *t*(97) = −0.24, *p* = .81), word prevalence (percentage of the population who know a particular word [*t*(77) = −0.11, *p* = .91], see Keuleers et al., 2015), and age of acquisition (the age at which a certain word was acquired [see Brysbaert et al., 2014], *t*(66) = 0.48, *p* = .64, see Table 1). WF and NA did not correlate (*r* = .02, *p* = .13).

**Table 1.** Maximum, minimum, means and standard deviations of word frequency (Zipf), name agreement, object recognition response time, word prevalence, age of acquisition, visual complexity and concept familiarity^a^ for old items (used in Session 1 and Session 2) and new items (used in Session 2 only).

Variable	Old				New			
	Max	Min	Mean	*SD*	Max	Min	Mean	*SD*
Name agreement (%)	100	24	72	19.23	100	25	72	18.65
Word frequency (Zipf)	4.93	1.66	3.35	0.86	4.98	1.66	3.38	0.81
OR RT (ms)	1769	220	613	239	1803	250	615	243
Word prevalence	1.96	1.08	1.85	0.13	1.96	0.88	1.84	0.13
Age of acquisition	11.72	3.71	6.74	1.63	11.78	3.92	6.56	1.51
Visual complexity	4.52	1.33	2.40	0.53	4.06	1.38	2.33	0.51
Concept familiarity	4.93	3.67	4.42	0.31	4.93	3.44	4.37	0.34

a Summarising statistics for visual complexity (VC) and concept familiarity (Fam) were added after running E1 and E2, upon Reviewer suggestion. VC refers to how complex or detailed a picture appears to be. Fam refers to how familiar participants are to the depicted object. Scores were taken from the original Canadian BOSS database (Brodeur et al., 2010, 2014) as such scores were not collected for speakers of Dutch (Decuyper et al., 2021).

*Note*. OR = object recognition; RT = response time.

In Session 1, each of the 98 pictures was presented three times, which means the session consisted of 294 experimental trials in total. Participants were assigned to one of four pseudorandomised lists (*n* = 24 or 25 per list). Items were never immediately preceded by a phonologically related item (starting with the same phoneme) or semantically related item (belonging to the same semantic category), and repetitions of the same item were separated by at least 20 trials. In Session 2, participants named all 98 *old* items presented in Session 1 and 98 *new* items they had not named before. Old and new items were pseudorandomised in such a way that items were not immediately preceded by a phonologically or semantically related item and no more than five old or new items were presented in consecutive trials. Participants were not familiarised with the pictures at the start of either session (see Table A2 of the Supplemental Material for a full list of items).

#### Procedure

Both sessions were administered online using Frinex (FRamework for INteractive EXperiments, a software package developed by the technical group at the Max Planck Institute for Psycholinguistics). Participants were instructed to complete the experiment in a quiet environment and to name pictures in Dutch while avoiding making any other sounds (such as ‘uhm’ or moving their chair). At the start of each session, participants were asked to check their microphone by creating a test recording. They were instructed to record a couple of seconds of speech (we suggested reading the instructions for the test recording if they did not know what else to say) and then listen to the audio playback. If there were any issues with the recording, they could check their audio settings and try again. Once they managed to make a clear test recording, they could continue to the experiment. Participants completed four practice trials prior to the main experiment.

Both sessions had an identical trial structure. Each trial started with a fixation cross (+) presented in the centre of the screen for 500 ms, followed by a 300 ms blank interval. Then the target picture was presented for 2,000 ms. If participants named the picture before the end of this 2,000 ms interval, they could press a ‘Next’ (‘Volgende’) button to continue. The next trial started 1,500 ms after button press or after time-out. Stimuli were preloaded at the start of the trial to ensure minimal delays in picture presentation. Participants were sent the link for Session 2 five to seven days after successful completion of Session 1.

### Data analysis

Naming latencies were measured from picture onset. Trained native Dutch speakers measured responses manually in Praat (Boersma & Weenink, 2001). Prior to the statistical analysis, we discarded 9% of all trials (*n* = 2,676) from Session 1 and 11% of trials (*n* = 2,055) from Session 2 for which the onset could not be measured due to poor audio quality or because participants did not respond within the 2,000 ms window, produced a disfluency, or gave an incorrect name that was not a plausible alternative to the modal name. Next, 3% (*n* = 691) of correct responses were removed from the data for Session 1 and 2% (*n* = 406) of correct responses in Session 2 because they were marked as outliers. Outliers were calculated as 2.5 *SD*s around the by-participant mean.

In our statistical analyses, we tested the effects of WF, NA and Repetition on naming latencies. We used linear mixed effects models (Baayen et al., 2008), specifically using the lme4 package (version 1.1-33, Bates et al., 2015) in RStudio version 4.3.0 (R Core Team, 2020). WF, NA and Repetition were treated as continuous variables; Set was contrast coded (old vs. new contrast coded as −1, 1). In an exploratory analysis, adding average response time for an item in the OR task (E1) as a fixed effect did not change the results and did not improve model fit (Akaike Information Criterion (AIC) and Bayesian Information Criterion (BIC) values were lower for the model without OR RTs). We therefore opted for the simpler models without OR RT. We used maximal random effect (Barr et al., 2013) without overfitting (i.e. models that yielded a singular fit warning were discarded).

We used NA and Zipf WF of the *given* response in all statistical models. In particular, we calculated NA for responses in the present study rather than using the NA value of a picture’s modal name based on the BOSS database (discussed in what follows) and used WF of the given response rather than the target response (*M*_BOSS_old_ = 3.34, *SD* = 0.86; *M*_BOSS_new_ = 3.37, *SD* = 0.81; *M*_data_old_ = 3.44, *SD* = 0.87; *M*_data_new_ = 3.62, *SD* = 0.84). Raw data, the analysis script, and full model structures and outputs are available at https://osf.io/b87qz/.

### Results

#### Correspondence between norms

To select our stimuli, we used values for NA from the BOSS database (Decuyper et al., 2021). BOSS data were collected in a written task with fewer participants (50–52 responses per picture). To test whether the norms for selected items generalise across modalities and samples, we calculated NA and *H*-values for NA in the current dataset and evaluated how well these variables correlate to the BOSS dataset. Before calculating NA and *H*, responses were aggregated across all responses pertaining to the same lemma (e.g. singular and plural or simple forms and diminutives were merged). NA was calculated as the percentage of participants who gave the most common response. The *H*-value is sensitive to the number of different names that were given to an object and the proportion of participants who used each of these names after excluding trials with no response (an object with a unique name [NA = 100%] has an *H*-value of 0). *H*-values for *old* items were calculated for responses at the first presentation of every item in Session 1; *H*-values for *new* items were based on data from Session 2.

NA (*R* = .60, *p* < .001) and *H*-values (*R* = .68, *p* < .001) in the BOSS database correlated moderately with those in our new dataset. More importantly, average NA for old and new items was almost identical in both datasets (*M*_BOSS_old_ = 72, *SD* = 19; *M*_BOSS_new_ = 72, *SD* = 19; *M*_data_old_ = 74, *SD* = 20; *M*_data_new_ = 74, *SD* = 18). The collected data showed that items had a distribution of NA similar to the one for stimuli selection based on BOSS. 84% of the items had the same modal name in the BOSS database and our dataset. We used the NA calculated in the present study in all further analyses. In particular, *actual* NA can especially be a better norm for determining modal names for items with low name agreement (see Corps & Meyer, 2025a).

### Session 1

#### All correct responses

**Figure 2.**
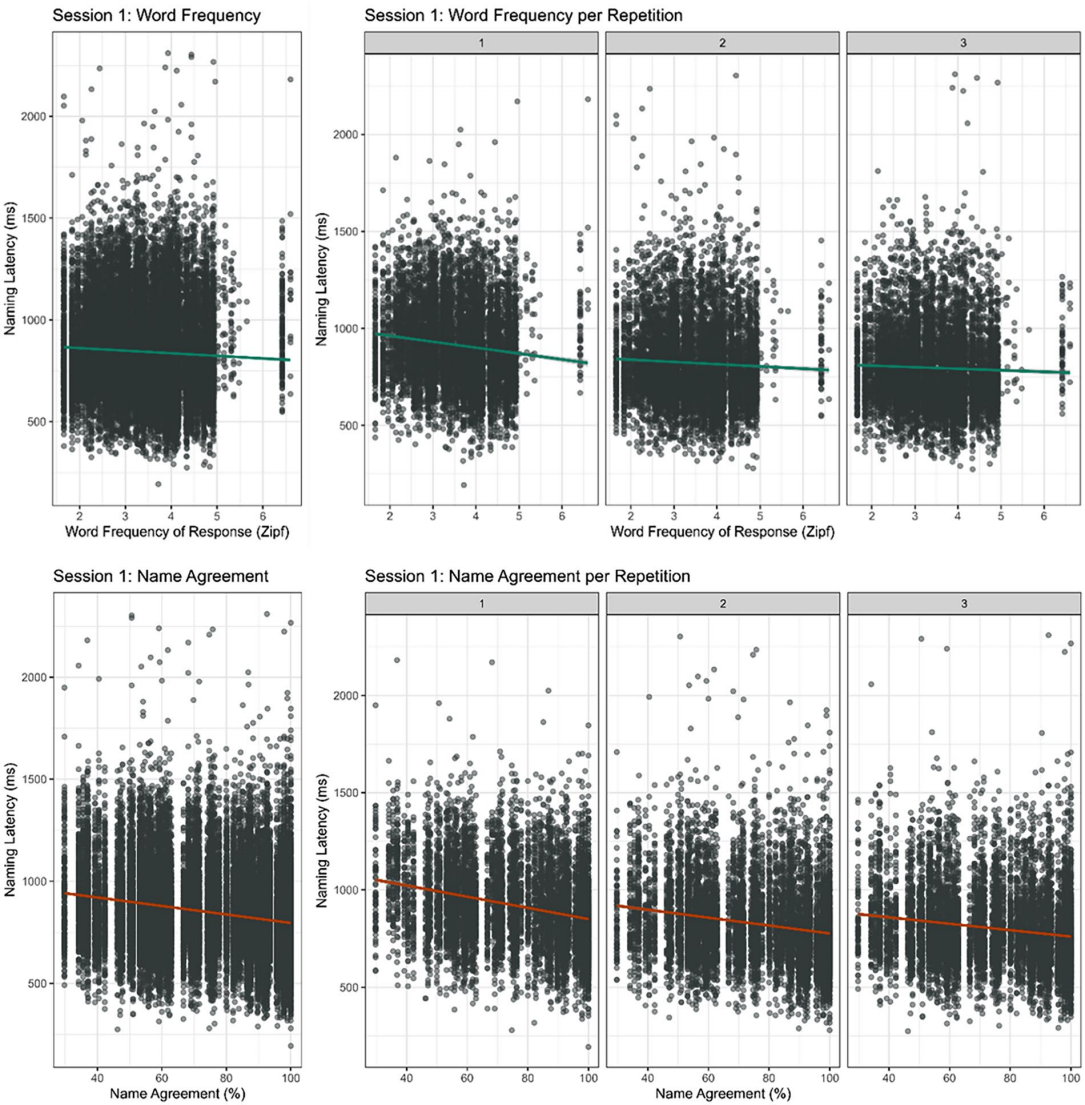
Naming latencies in Session 1 of the online picture naming task by word frequency (top) and name agreement (bottom); overall (left) and per repetition (right). Plots contain all correct responses to all pictures.

In the model including all correct responses in Session 1, naming latencies were predicted by the interaction between Repetition and NA and the interaction between Repetition and WF of the response as main effects and by-subject and by-item intercepts as random effects.^2^ As mentioned before, we used Zipf WF of the actual response and calculated NA for the responses in this study rather than using frequency and NA of the modal name selected from the BOSS database. On average, participants responded 847 ms (*SD* = 216) after picture onset. Participants’ naming latencies decreased with each repetition (β = −132.58, *t* = −19.93, *p* < .001; r1 *M* = 919 ms, *SD* = 217; r2 *M* = 827 ms, *SD* = 210; r3 *M* = 802 ms, *SD* = 205). The model revealed main effects of WF (β = −28.49, *t* = −7.85, *p* < .001) and NA (β = −3.16, *t* = −10.17, *p* < .001), indicating faster responses for items with high WF compared to items with low WF and faster responses for items with high NA compared to items with low NA respectively (see Figure 2). The model further revealed significant interactions between Repetition and WF (β = 9.22, *t* = 6.09, *p* < .001) and Repetition and NA (β = .52, *t* = 7.67, *p* < .001), indicating both the NA and WF effects can be affected by repetition.

To assess the effects of WF and NA per repetition, we ran a model that included main effects of WF and NA and by-participant and by-item intercepts in its random effects structure on the same data, now split by repetition. Participants were faster to name items with high WF compared to items with low WF on the first two presentations (r1: β = −18.33, *t* = −5.46, *p* < .001; r2: β = −8.48, *t* = −2.80, *p* < .01; r3: β = −4.70, *t* = −1.60, *p* = .11). They were faster to name high NA items compared to low NA items on every repetition (r1: β = −2.83, *t* = −8.00, *p* < .001, r2: β = −1.95, *t* = −6.99, *p* < .001, r3: β = −1.67, *t* = −5.86, *p* < .001). The effect of WF attenuated more across repetitions than the effect of NA, suggesting that, within Session 1, the WF effect was affected more by repetition priming than the NA effect.

#### Consistent responses

The next analysis focuses on consistent responses only. By consistent, we mean identical responses with the same morphological form (e.g. *boek* and diminutive *boekje* were coded as different responses). We expect a larger effect of repetition when participants repeatedly use the same name for an object (and thus go through the same word retrieval procedure) throughout the experiment. We ran the same statistical models as described above on a subset of the data. This dataset kept 71% of datapoints compared to the dataset including all correct responses. Using the same name throughout Session 1 sped up RTs (*M* = 816 ms, *SD* = 204). Again, the model showed main effects of WF (β = −35.28, *t* = −8.47, *p* < .001) and NA (β = −3.10, *t* = −10.10, *p* < .001). Participants responded faster with every repetition (β = −174, *t* = −23.34, *p* < .001; r1 *M* = 901 ms, *SD* = 208; r2 *M* = 783 ms, *SD* = 187; r3 *M* = 765 ms, *SD* = 190). The model again revealed significant interactions between Repetition and WF (β = 13.45, *t* = 7.95, *p* < .001) and Repetition and NA (β = .76, *t* = 10.06, *p* < .001). Participants were only faster to name items with high WF compared to items with low WF on the first presentation (r1: β = −30.72, *t* = −7.41, *p* < .001; r2: β = 3.72, *t* = 1.02, *p* = .31; r3: β = 1.19, *t* = 0.32, *p* = .75). The NA effect was more robust. Participants were faster to name high NA items compared to low NA items on all repetitions (r1: β = −2.58, *t* = −7.22, *p* < .001; r2: β = −1.13, *t* = −4.17, *p* < .001; r3: β = −1.01, *t* = −3.63, *p* < .001). The difference in effect of repetition on WF and NA is more clearly visible when speakers consistently use the same name for a picture than when they switch names. One repetition was enough to eliminate the WF effect; the NA effect remains significant after three repetitions. It thus seems that the WF effect is more susceptible to the effects of repetition than the NA effect.

#### Modal responses

Onset latencies of responses in which participants used the (within this study) modal name (76% of the data, *M* = 827 ms, *SD* = 209) showed a very similar pattern to the previous analyses. The WF effect was only significant on the first presentation, and the NA effect remained significant on all repetitions. To explore the difference between modal names and alternative names, we ran a model with a subset of items with NA lower than 70% as calculated in the current dataset (i.e. items that clearly have multiple plausible names). In this subset of correct responses (11,041 datapoints), speakers used the modal name in 62% (and a correct alternative name in 38%) of trials. Latencies were predicted by response type (modal name or correct alternative name; contrast coded as −1 and 1). Random effects consisted of intercepts for subject and item. The model revealed a significant difference between naming latencies for both response types (β = 19.15, *t* = 10.18, *p* < .001). Participants responded faster when they used the modal name for a picture compared to when they used a correct alternative name (*M*_modal_ = 856 ms, *SD*_modal_ = 215; *M*_alt_ = 908, *SD*_alt_ = 226). Even when participants can use whichever name they prefer, they still seem to be aware of the modal name for a particular item. When using an alternative name, they still consider other options, resulting in longer naming latencies.

### Session 2

To assess whether any effects of repetition were long-lasting, we compared old and new items named in Session 2, which was administered 5 to 7 days after Session 1.

#### All correct responses

**Figure 3.**
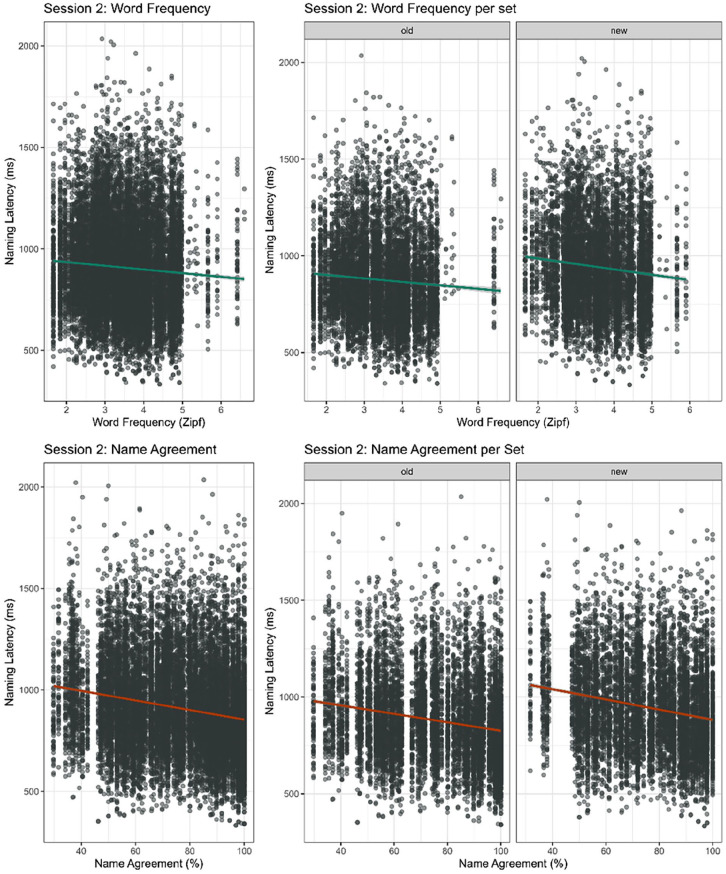
Naming latencies in Session 2 of the online picture naming task by word frequency (top) and name agreement (bottom); overall (left) and per set (right). Plots contain all correct responses to all pictures.

First, we ran a model on all correct responses in Session 2. Naming latencies were predicted by the interaction between Set (old items vs. new items) and NA, and the interaction between Set and WF of the response. Random effects consisted of by-subject and by-item intercepts. On average, participants responded 912 ms (*SD* = 228) after picture onset. The model showed main effects of WF (β = −16.71, *t* = −7.09, *p* < .001) and NA (β = −2.39, *t* = −8.66, *p* < .001), replicating faster responses for items with high WF compared to items with low WF and faster responses for items with high NA compared to items with low NA, respectively. There was no interaction between WF and Set (β = −1.70, *t* = −0.74, *p* = .46), or between NA and Set (β = −.22, *t* = 0.79, *p* = .43. Any attenuation of effect of WF or NA found in Session 1, did not affect naming in Session 2 (Figure 3). The model did show an overall effect of Set (β = 59.18, *t* = 2.70, *p* < .01), indicating there was an overall practice effect of naming pictures in Session 1 (*M*_old_ = 880, *SD* = 233; *M*_new_ = 947, *SD* = 233).

In an additional analysis, we compared response times for the *same* items on their final (third) presentation in Session 1 and on their presentation as old items in Session 2. Naming latencies were predicted by the interaction between WF and Session (S1 r3 vs. S2 old) and the interaction between NA and Session. Random effects consisted of by-subject and by-target intercepts. The model showed interactions between WF and Session (β = 3.01, *t* = 2.02, *p* < .05) and NA and Session (β = −0.29, *t* = 4.32, *p* < .001), confirming that the WF and NA effects were weaker at the end of Session 1 (WF: β = −4.71, *t* = −1.60, *p* > .05; NA: β = −1.67, *t* = −5.86, *p* < .001) compared to in Session 2 (WF: β = −15.99, *t* = −5.02, *p* < .001; NA: β = −2.17, *t* = −6.35, *p* < .001). These results statistically confirm that the WF effect disappeared and the NA effect attenuated by the end of Session 1, but both regained strength in Session 2, indicating repetition did not have long-lasting effects on the effects of WF and NA.

#### Consistent responses

We ran the same model to compare WF and NA effects on ‘old’ and ‘new’ items. For old items however, we only included responses that were consistent between all four occurrences of the item in Session 1 and Session 2 (13,545 datapoints). Participants were slightly faster when they used the same names in Session 1 as in Session 2 (*M* = 902 ms, *SD* = 226) compared to when they used any correct name. The model revealed main effects of WF (β = −14.36, *t* = −4.91, *p* < .001) and NA (β = −2.20, *t* = −8.19, *p* < .001), confirming faster responses for items with high WF compared to items with low WF and faster responses for items with high NA compared to items with low NA. There was no interaction between WF and Set (β = −4.07, *t* = −1.42, *p* = .16), or between NA and Set (β = −.40, *t* = −1.49, *p* = .14). This means that even though the WF effect and NA effect in Session 1 were eliminated after one repetition or reduced within the session, respectively, these changes in effect were not long-lasting. The three repetitions in Session 1 were not enough to alter a word’s accessibility in Session 2. The model did report an overall effect of set (β = 93.53, *t* = 4.25, *p* < .001), again showing a practice effect for pictures that had been named in Session 2.

To compare items that were named the same way in both Session 1 (on their third presentation) and Session 2 (old items), we ran the same exploratory analysis as described for the dataset with all correct responses, using the same statistical model. Here, the model revealed an interaction between Session and NA (β = .42, *t* = 5.44, *p* < .001), again confirming the NA effect was weaker at the end of Session 1 (β = −1.01, *t* = −3.63, *p* < .001) compared to in Session 2 (β = −1.79, *t* = −5.69, *p* < .001). However, there was no interaction between Session and WF (β = 2.01, *t* = 1.16, *p* > .05). These results show that, for a subset of responses, the WF effect did not regain strength between the end of Session 1 and Session 2. Repetition thus seems to have a more long-lasting effect (at least up to 1 week later) on the WF effect when speakers used the same name (i.e. when they got the maximum benefit from practicing).

#### Modal responses

A separate analysis with onset latencies of responses in which participants used the (within this study) modal name (76% of the data, *M* = 888 ms, *SD* = 222, same model as used for Session 1) showed no main effect of WF (*p* = .33) and a significant effect of NA (*p* < .001). However, there was no effect of Set and there were no interactions between Set and NA or WF, so we cannot conclude the WF effect disappeared in Session 2 because of repetition effects in Session 1. As for Session 1, we compared naming latencies for modal names and correct alternative names in a model with all correct responses for a subset of pictures with low NA (<70%; 16,948 datapoints). Again, naming latencies were shorter for modal names (76% of responses) compared to naming latencies for alternative names (*M*_modal_ = 919 ms, *SD*_modal_ = 229; *M*_alt_ = 989, *SD*_alt_ = 228). Using the same model as for the data from Session 1, we found this difference to be significant (β = 26.71, *t* = 11.37, *p* < .001). Replicating results from Session 1, this supports the idea that even when participants are free to choose an object name, they still seem to be aware of community knowledge about the preferred name for a certain object when they select one.

## General discussion

In this two-session picture naming study, we investigated whether WF and NA effects were influenced by-item repetition. In Session 1, participants were faster to name high-frequency compared to low-frequency items on their first and second presentation, although this word frequency effect was reduced on the second presentation compared to the first repetition. When speakers used the same name on all repetitions (71% of data), and thus went through the same retrieval process on each repetition, the WF effect disappeared after one repetition. The NA effect was also reduced by repetition, but participants were still faster to name high NA compared to low NA pictures on all repetitions, even when they used the same names throughout the experiment. Thus, the word frequency effect was attenuated more by repetition than the NA effect. None of the changes in effects in Session 1 were still detected in Session 2 (5–7 days after Session 1). There was no difference in the size of the WF or NA effect between *old* items that had been named in Session 1 and *new* items that were only presented in Session 2. Therefore, our study shows that previous use of a word can affect this word’s accessibility within one experimental session (i.e. short-term), but does not provide evidence that this effect is long-lasting.

### Repetition reduces WF and NA effects within Session 1

Our study shows that the WF effect was reduced in the course of one experimental session, thus replicating previous findings (Corps & Meyer, 2023, 2025b; Griffin & Bock, 1998). This pattern is consistent with the assumption that the structure of the mental lexicon is flexible and can change with experience (Damian & Als, 2005; Howard et al., 2006; Oppenheim et al., 2010). Naming a picture strengthens the link between the depicted concept and the selected name. This learning episode makes the word’s representation more accessible by leaving a trace in memory. Repeated exposure is especially beneficial to low-frequency items, as high-frequency items already have stronger links between their concepts and names. The change in accessibility of low WF items is what reduces the WF effect.

The NA effect was also reduced within Session 1. Again, the strengthening of the link between a concept and a name over multiple repetitions can explain this attenuation in the NA effect. Low NA items have multiple plausible names. Repeated use of one name creates a bias towards that name by strengthening its links to the concept and weakening the links to unselected names, and as a result, reduces the competition between alternatives (see also Wöhner et al., 2024). Unlike the WF effect, the NA effect was not completely eliminated by repetition. This finding replicates a finding in a study by Corps and Meyer (2025a), where the NA effect was also reduced but not eliminated after familiarisation and three repetitions of the items. One account of this pattern is that three repetitions are enough to change the accessibility of low NA items. It might simply take more time to eliminate the NA effect compared to the WF effect.

Alternatively, the NA effect might also be too robust to eliminate regardless of the number of repetitions. Speakers might still represent other plausible names for an object even after they have used one name repeatedly, and so the competition between good alternatives might remain strong. In addition to this competition between multiple names for an item that a speaker might find appropriate (‘individual NA’), speakers are also thought to represent how other speakers would name an object (‘population-level NA’). A study by Balatsou et al. (2022) showed that when speakers named high and low NA pictures twice, they tended to use the same name on both occasions. But when they used different names, they were more likely to switch from a name that was relatively uncommon in the community to a more commonly used one than doing the reverse (i.e. replacing a commonly used name by a less common one). These findings indicate that even though speakers might have personal preferences, population-level NA (i.e. how other speakers might name a picture) is also represented in the individual speakers’ minds. This explanation is supported by our response time analysis of modal versus alternative names. For objects with low NA (<70%), RTs for modal names were faster compared to alternative names, even though the task allowed participants to respond with whichever name they preferred. This suggests that speakers have some knowledge about the modal names of the items within their community. When they choose different names, these modal names remain potent competitors, even after several repetitions of the items.

In sum, repetition can affect a word’s accessibility and modulate the effects of WF and NA short-term (within Session 1). The WF effect seems to attenuate more than the NA effect, especially when speakers consistently use (and practice) the same name, but, as we did not statistically compare the effect sizes, we only have qualitative evidence for this difference. The required analysis would be challenging as the variables are measured on different scales.

### Long-term effects of repetition

We set out to assess whether any attenuation of the WF and NA effect in Session 1 would still be detectable in Session 2, taking place 5 to 7 days after Session 1. We found that in Session 2, participants were overall faster to name old items (which they had named before) than new items. We also saw a WF and an NA effect in Session 2. Moreover, we did not find a difference in the strength of these effects for old items and new items. Therefore, participants had some memory of the items named before, but the strength of the WF and NA effect was not altered.

This pattern of results suggests that naming the pictures in Session 1 affected the participants’ memory for the pictures and their names in at least two ways, namely at the visual-conceptual and lexical level. Seeing and naming the pictures facilitated the recognition of the depicted objects. This effect was strong and long-lived and was probably the main contributor to the between-session repetition effect. This proposal is consistent with multiple earlier studies showing (very) long-lasting memory for pictures (e.g. Cave, 1997; Mitchell & Brown, 1988), and with studies that determined the origins of repetition effects in picture recognition, naming and translation tasks (e.g. Francis et al., 2008; Francis & Sáenz, 2007; Lachman et al., 1980). It is also consistent with our earlier finding (Corps & Meyer, 2025b) that the between-sessions repetition priming effect for picture naming was stronger when participants had named the pictures in Session 1 than when they read the picture names and conducted a semantic categorisation task. Clearly, the early visual-conceptual processes involved in picture naming benefit strongly from repetition priming.

Turning to the lexical level, our results showed that naming affected a word’s accessibility within Session 1. As discussed above, this result can be explained within contemporary models of lexical learning (e.g. Damian & Als, 2005; Howard et al., 2006; Oppenheim et al., 2010). Using a word to name a picture strengthens the links between the relevant conceptual and lexical representations and may also weaken links between the conceptual and lexical representations of activated, but not select, alternative names. These changes in the lexical network facilitate using the name again when the picture is repeated, in particular for ‘harder’ items, that is, in our case, items with lower frequency names or lower name agreement. Our results suggest that these changes are short-lived, as the WF and NA effects reappear in Session 2. A straightforward account of this result is that the weight changes decay over time, as typically assumed in theories of lexical learning (e.g. Oppenheim et al., 2010).

To the best of our knowledge, there are no other studies that have investigated whether repetition has a longer-term effect on the size of the NA effect. But our findings concerning the WF effect can be compared to those reported by Corps and Meyer (2025b). In Session 1 of that study, participants named pictures six times each. This eliminated the WF effect within this session as well as in Session 2. In that session, a new set of items was introduced, which was only named once and then tested again in Session 3. For this item set, the frequency effect was still seen in Session 3. A conclusion to be drawn from both studies is that the frequency effect can be reduced longer term, provided that there is a sufficient number of naming episodes within a short period of time. Naming pictures once, or three times, does not yield a durable attenuation of the word frequency effect, but naming them six times does.

A speculative account of this pattern within a model of lexical learning is that item repetition within a session counteracts the decay of weight changes described above (Corps & Meyer, 2025b). Thus, naming a picture temporarily strengthens the connections between the concept invoked by the picture and the lexical representation of the picture name. The decay of these changes is counteracted when a picture is named again within a short time span. When there are multiple encounters of a word within a short time span, the strengthening of the connections appears to outweigh the decay, leading to stronger and more durable changes in the lexical network.

Further work is needed to understand how short-term and longer-term lexical learning occur. For instance, in our study, repetition attenuated both the word frequency and, to a lesser degree, the NA effect. This pattern does not allow us to draw strong conclusions about the specific ways repetition affects access to a single low-frequency name of a picture, or access to one of the names of a picture with multiple plausible names. More experimental and computational work is needed to map out how different properties of the lexical network are changed through learning. Similarly, in our paradigm, longer-term lexical learning was only observed after more than three learning episodes within a session. This result is likely specific to the picture naming paradigm, as children and adults evidently learn words from reading and hearing them in a variety of contexts. A broad question is how properties of a learning episode (such as the spacing of items or the learner’s task) change specific aspects of lexical representations during this learning episode. Finally, there is strong evidence that changes in lexical representations can be consolidated off-line, possibly during sleep (e.g. Gaskell et al., 2019; Rodd et al., 2013, 2016). An important area of investigation is how changes in lexical representations during a learning episode and consolidation processes jointly shape the structure of the mental lexicon.

### Methodological implications: BOSS norms

This study allowed us to compare written norms for NA as reported in the BOSS database to NA percentages in the current dataset with spoken responses. NA and *H*-values correlated reasonably well between written and spoken norms, the average NA of items used in this study was the same, and 84% of the items had the same modal name in both datasets (70% for items with NA lower than 70). There was some variation in both NA percentages and modal names. This variation could be explained by differences in samples and sample sizes. In the BOSS norming study, pictures were named by participants in the lab, drawing from the usual pool of university students who were all located in the same city. The online study was mostly administered through Prolific, allowing for more variation in speakers in terms of, for instance, education or where they live in the Netherlands. If NA is based on community-based knowledge, NA might have differed between a more homogeneous population in the BOSS study and the group of speakers in the current study. Furthermore, in the current study, pictures were named by twice as many participants, again allowing for more variation.

Another explanation for the differences in NA between the two datasets could be the difference in tasks. First, BOSS norms were calculated from written responses, whereas the present study collected spoken responses. Although we do not expect these responses to differ a lot in naming common objects, we noticed that for some items, the modal spoken name was shorter or slightly less precise than the modal written name (e.g. *mes [knife]* instead of *broodmes [bread knife]; fiets [bike]* instead of *mountainbike*). Second, or alternatively, this difference in response length or specificity could be explained by the lack of time pressure in the BOSS study. Despite this variation in modal names for a subset of items in this study (16% of all items; 30% of low NA items), the BOSS norms remain a good estimate of how many different names an item could get when used in written or spoken picture naming studies. Norms for spoken responses, collected in this study, will have been added to the BOSS database on the OSF: https://osf.io/kwu87/.

## Conclusions

In conclusion, we tested how repetition moderated the effects of word frequency and name agreement in a picture naming task. Within a test session, both effects were reduced across the three presentations of the materials, with the reduction being descriptively more pronounced for word frequency than name agreement. Contrary to earlier findings, both effects reappeared in Session 2. Further research is necessary to elucidate under which conditions using words leads to memory traces that are durable enough to lead to longer-term changes in the structure of our mental lexicon.

## Supplemental Material

sj-docx-1-qjp-10.1177_17470218251365517 – Supplemental material for Repetition leads to short-term reduction of word frequency and name agreement effects: Evidence from a Dutch two-session picture naming experiment Supplemental material, sj-docx-1-qjp-10.1177_17470218251365517 for Repetition leads to short-term reduction of word frequency and name agreement effects: Evidence from a Dutch two-session picture naming experiment by Caitlin Decuyper, Ruth E Corps and Antje S Meyer in Quarterly Journal of Experimental Psychology

## Notes

1. In a post-hoc exploratory analysis (upon Reviewer suggestion), we added concept familiarity (Fam) and visual complexity (VC, see Table 1) as covariates to the model. There were no effects of Fam or VC and including these variables did not improve model fit.

2. In an exploratory analysis (upon Reviewer suggestion), we added concept familiarity and visual complexity (see Table 1) as covariates to the model. Adding these variables did not affect significance of effects or interactions and did not improve model fit.
