# Supplementary material for: Repetition leads to short-term reduction of word frequency and name agreement effects: Evidence from a Dutch two-session picture naming experiment
Source: Q J Exp Psychol (Hove). 2025 Aug 2;79(4):803–18. doi: 10.1177/17470218251365517 (PMC12982551; doi:10.1177/17470218251365517)
Supplement: sj-docx-1-qjp-10.1177_17470218251365517 – Supplemental material for Repetition leads to short-term reduction of word frequency and name agreement effects: Evidence from a Dutch two-session picture naming experiment [file sj-docx-1-qjp-10.1177_17470218251365517.docx]

**Supplementary Materials for:**

**Repetition leads to short-term reduction to word frequency and name agreement effects: Evidence from a Dutch two-session picture naming experiment**

Caitlin Decuyper^1*^, Ruth E. Corps^2,1^, & Antje S. Meyer^1,3^

^1^Max Planck Institute for Psycholinguistics, Nijmegen, the Netherlands

^2^University of Sheffield, Sheffield, United Kingdom

^3^Donders Institute for Brain, Cognition, and Behaviour, Radboud University

*Corresponding author at: Max Planck Institute for Psycholinguistics, PO Box 310, 6500 AH Nijmegen, The Netherlands. E-mail address: [Caitlin.Decuyper@mpi.nl](mailto:Caitlin.Decuyper@mpi.nl)

| **Table A1**  Experimental and filler items used Experiment 1 (Object Recognition). For experimental items, the word preceding the picture was always the picture’s name. For filler items, the word did not match the picture’s name. | | |
| --- | --- | --- |
| Experimental items | Filler items | |
| Picture/word | Picture | Word |
| aap | aanhangwagen | reiger |
| aluminiumfolie | aardappel | motor |
| arend | aardbei | sandaal |
| autosleutel | accordeon | pijp |
| bagel | achteruitkijkspiegel | rijst |
| band | afstandsbediening | meetlint |
| bankje | afwasmiddel | hagedis |
| been | anker | roltrap |
| beer | antenne | verkeersbord |
| beker | appel | keukenrol |
| bijl | artisjok | klarinet |
| bladblazer | augurk | skelet |
| blik | baksteen | drumstokken |
| blokfluit | bakvorm | zeehond |
| boomstam | balkon | stier |
| boot | ballon | surfplank |
| bord | barbecue | lippenstift |
| borstel | basket | handdoek |
| boterham | batterij | klavertje |
| bril | beitel | cassettebandje |
| brood | bel | laptop |
| broodmes | bezem | kalender |
| broodrooster | bierdopje | anker |
| bureaustoel | bierflesje | elleboog |
| bus | bijenkorf | kapstok |
| cabrio | biljartbal | otter |
| cactus | bleekselderij | eikel |
| cadeau | bloem | antenne |
| camera | bloemkool | feesthoedje |
| cap | bloempot | loopband |
| chips | boeddha | snowboard |
| cupcake | bokshandschoen | pijl |
| dartbord | boog | matras |
| dinosaurus | boom | zandloper |
| doos | botsauto | dienblad |
| douchekop | brandkraan | zeester |
| driewieler | brievenbus | aanhangwagen |
| dromenvanger | broek | paprika |
| druiven | buis | zebra |
| duif | cassettebandje | gordijn |
| duikbril | cd | douchecabine |
| eieren | cello | zonnebril |
| fietspomp | champagne | zeef |
| fotolijstje | cheeta | pad |
| friet | computer | schouder |
| geweer | container | draad |
| giraffe | courgette | grasmaaier |
| gitaar | croissant | baksteen |
| gloeilamp | cruiseschip | achteruitkijkspiegel |
| haai | deuren | cello |
| haarclip | deurklink | botsauto |
| haarspeld | dienblad | tasje |
| hamer | discobal | lolly |
| handschoen | dobbelsteen | augurk |
| hek | dolfijn | perforator |
| helm | douchecabine | rits |
| hoed | draad | vaas |
| hoefijzer | dromedaris | schoolbord |
| hoepel | drumstokken | koffieautomaat |
| hondenhok | eekhoorn | legoblokje |
| honkbal | eikel | schommel |
| horloge | eisnijder | viool |
| huisnummer | elastiek | peddel |
| ijshoorntje | elleboog | boeddha |
| jetski | feesthoedje | roos |
| kaarsje | fietsslot | kampvuur |
| kaart | flesje | deurklink |
| kandelaar | flesopener | joker |
| kast | flosdraad | voetbaltafel |
| kasteel | framboos | zwembroek |
| kat | gember | spatel |
| kegel | gier | kompas |
| kentekenplaat | golfbal | boog |
| ketting | gordijn | krab |
| kinderwagen | graafmachine | bokshandschoen |
| kleerhanger | graan | ballon |
| kliko | gradenboog | ijsbeer |
| knoflook | grafsteen | tuinslang |
| koffiebonen | grasmaaier | pizza |
| koffiekan | haan | golfbal |
| koffiezetapparaat | haarband | kabel |
| kopje | hagedis | eisnijder |
| krijtje | hakmes | videoband |
| kroon | handboeien | bakvorm |
| kruis | handdoek | kerstboom |
| kussen | havermout | brievenbus |
| kwast | helikopter | grafsteen |
| laars | honkbalknuppel | picknicktafel |
| lamp | ijsbeer | wolk |
| lampjes | ijskrabber | schedel |
| lantaarnpaal | jeep | computer |
| lepel | joker | schaap |
| lieveheersbeestje | juskom | cruiseschip |
| lp | kaas | handboeien |
| luier | kabel | tulp |
| maan | kakkerlak | veter |
| maiskolf | kalender | broek |
| mandje | kampvuur | pen |
| map | kanon | paraplu |
| masker | kapstok | ijskrabber |
| meeuw | kattenbak | statief |
| megafoon | kerstbal | juskom |
| mes | kerstboom | spuitbus |
| mixer | keukenrol | lucifer |
| mok | keyboard | schoorsteen |
| mountainbike | kinderfiets | gember |
| muis | kip | discobal |
| munten | kiwi | flesopener |
| naaktslak | klarinet | hakmes |
| nagelknipper | klavertje | triangel |
| nietjes | kluis | lippen |
| nietmachine | knie | appel |
| nijlpaard | koe | vliegtuig |
| nintendo | koekje | bel |
| olifant | koelkast | havermout |
| oog | koffieautomaat | aardappel |
| ooglapje | kokosnoot | buis |
| oor | komkommer | scalpel |
| overhemd | kompas | strijkplank |
| paard | koptelefoon | slang |
| pan | krab | wasmand |
| panda | krokodil | rookmelder |
| pantoffel | ladder | kiwi |
| penne | laptop | framboos |
| pepermolen | legoblokje | bierflesje |
| pet | limoen | wattenstaafje |
| pinautomaat | liniaal | knie |
| piratenvlag | lippen | dolfijn |
| plakband | lippenstift | accordeon |
| plank | lolly | kanon |
| plant | loods | afwasmiddel |
| podium | loopband | vingerafdruk |
| pompoen | lucifer | maatbeker |
| pooltafel | lynx | balkon |
| poort | maatbeker | kakkerlak |
| pop | macaroni | pauw |
| prikbord | magnetron | vlinder |
| printer | mango | touw |
| prullenbak | mascara | spijker |
| puzzelstukje | matras | lynx |
| reddingsvest | meetlint | paperclip |
| riem | motor | stift |
| rietje | mueslireep | toetsenbord |
| rugzak | muizenval | aardbei |
| schaakbord | muts | pruim |
| schaal | naaimachine | jeep |
| schaats | ontstopper | bijenkorf |
| schakelaar | orka | bloem |
| schelp | otter | muts |
| schep | ovenschaal | basket |
| schilderij | pad | brandkraan |
| schildpad | paperclip | tang |
| schoen | paprika | scheerapparaat |
| schoolbus | paraplu | koptelefoon |
| schort | parasol | container |
| schrift | pauw | batterij |
| shirt | peddel | schoffel |
| skibril | peer | mascara |
| sla | pen | kokosnoot |
| sleutelbos | perforator | rozijnen |
| slipper | picknicktafel | cd |
| slot | pijl | limoen |
| sneeuwpop | pijp | gradenboog |
| snoepje | pizza | flosdraad |
| soeplepel | plantenspuit | kluis |
| sok | portemonnee | kattenbak |
| spaarpot | potlood | tuinhuisje |
| speen | prijskaartje | biljartbal |
| spiegel | pruim | vergiet |
| spijkerbroek | puntenslijper | gier |
| spin | radio | peer |
| spinnenweb | reiger | kinderfiets |
| springtouw | rijst | bezem |
| spuit | ring | parasol |
| startkabels | rits | puntenslijper |
| stoel | roltrap | koekje |
| stokbrood | rookmelder | haarband |
| stopcontact | roos | step |
| stoplicht | rozijnen | windwijzer |
| strijkijzer | sandaal | ladder |
| strik | scalpel | bloempot |
| stropdas | schaap | prijskaartje |
| taart | schedel | croissant |
| tablet | scheerapparaat | toeter |
| tafel | schoffel | naaimachine |
| tas | schommel | plantenspuit |
| teddybeer | schoolbord | elastiek |
| tegels | schoorsteen | vulpen |
| telefooncel | schouder | telescoop |
| tennisracket | schroevendraaier | kaas |
| theepot | sigaar | muizenval |
| tijger | sinaasappel | krokodil |
| tissues | skelet | vos |
| tomaten | slang | flesje |
| trampoline | snowboard | mueslireep |
| trap | spatel | komkommer |
| trapleuning | speelgoedauto | magnetron |
| t-shirt | spijker | bloemkool |
| tuinkabouter | spuitbus | waterlelie |
| tv | statief | courgette |
| ui | step | beitel |
| uil | stier | graan |
| usb-stick | stift | thermometer |
| varken | strijkplank | radio |
| veer | surfplank | kerstbal |
| veerboot | sushi | keyboard |
| veiligheidsbril | tang | honkbalknuppel |
| veiligheidsspeld | tasje | dobbelsteen |
| verfblik | telescoop | boom |
| vergrootglas | thermometer | sinaasappel |
| verwarming | toeter | barbecue |
| videocamera | toetsenbord | wiel |
| vingerhoedje | toren | volleybal |
| vishengel | touw | zonneklep |
| vlag | triangel | sigaar |
| vlieger | tuinhuisje | helikopter |
| voet | tuinslang | cheeta |
| voetbal | tulp | dromedaris |
| voetbalschoen | vaas | loods |
| vogel | vergiet | portemonnee |
| vogelbekdier | verkeersbord | eekhoorn |
| vogelnest | veter | liniaal |
| vork | videoband | potlood |
| vrachtwagen | vingerafdruk | orka |
| waaier | viool | artisjok |
| wasbak | vliegtuig | macaroni |
| wasknijper | vlinder | fietsslot |
| waterflesje | voetbaltafel | ontstopper |
| watermeloen | volleybal | kip |
| waterput | vos | speelgoedauto |
| wc | vulpen | mango |
| weegschaal | wasmand | sushi |
| wereldbol | waterlelie | bierdopje |
| wereldkaart | wattenstaafje | deuren |
| wijn | wiel | koe |
| wijnglas | windwijzer | zwijn |
| windmolen | wolk | schroevendraaier |
| winkelwagen | zandloper | koelkast |
| wol | zebra | champagne |
| wolf | zeef | toren |
| wortel | zeehond | ovenschaal |
| zaag | zeester | haan |
| zandkasteel | zonnebril | graafmachine |
| zeilboot | zonneklep | afstandsbediening |
| zoutvaatje | zwembroek | bleekselderij |
| zwaard | zwijn | ring |

| **Table A2**  Old items and New items used in Experiment 2 (Picture Naming). Old items were presented three times in Session 1 and presented once in Session 2; New items were presented once in Session 2 | |  |
| --- | --- | --- |
| Old items | New items |  |
| autosleutel | aap |  |
| bagel | aluminiumfolie |  |
| blokfluit | arend |  |
| boterham | band |  |
| broodmes | bankje |  |
| broodrooster | been |  |
| cactus | beer |  |
| cupcake | bijl |  |
| doos | boomstam |  |
| douchekop | boot |  |
| druiven | borstel |  |
| fietspomp | bureaustoel |  |
| geweer | bus |  |
| gloeilamp | cabrio |  |
| haai | cap |  |
| haarclip | chips |  |
| hamer | dinosaurus |  |
| hoepel | driewieler |  |
| honkbal | duif |  |
| huisnummer | duikbril |  |
| kaart | eieren |  |
| kast | fotolijstje |  |
| kleerhanger | friet |  |
| knoflook | gitaar |  |
| koffiekan | handschoen |  |
| koffiezetapparaat | helm |  |
| kopje | horloge |  |
| kroon | ijshoorntje |  |
| kruis | jetski |  |
| lepel | kandelaar |  |
| lp | kat |  |
| maiskolf | kegel |  |
| meeuw | kentekenplaat |  |
| megafoon | kinderwagen |  |
| mixer | kliko |  |
| mountainbike | krijtje |  |
| muis | kussen |  |
| olifant | kwast |  |
| oog | laars |  |
| ooglapje | lampjes |  |
| overhemd | lantaarnpaal |  |
| paard | luier |  |
| pan | map |  |
| pantoffel | mok |  |
| penne | munten |  |
| pepermolen | naaktslak |  |
| pet | nietmachine |  |
| pinautomaat | Nintendo |  |
| plakband | panda |  |
| plant | piratenvlag |  |
| pompoen | plank |  |
| printer | pooltafel |  |
| reddingsvest | poort |  |
| rugzak | prikbord |  |
| schaal | prullenbak |  |
| schakelaar | riem |  |
| schelp | rietje |  |
| schilderij | schaakbord |  |
| schildpad | schaats |  |
| schoen | schep |  |
| schort | schoolbus |  |
| schrift | sla |  |
| shirt | sleutelbos |  |
| slipper | slot |  |
| snoepje | sneeuwpop |  |
| speen | sok |  |
| spiegel | spaarpot |  |
| spin | spijkerbroek |  |
| spinnenweb | stokbrood |  |
| spuit | strijkijzer |  |
| stoel | strik |  |
| stoplicht | stropdas |  |
| taart | tablet |  |
| tas | tafel |  |
| teddybeer | tegels |  |
| theepot | telefooncel |  |
| trampoline | tennisracket |  |
| tuinkabouter | tijger |  |
| tv | tissues |  |
| ui | tomaten |  |
| usb-stick | trap |  |
| veer | trapleuning |  |
| verwarming | t-shirt |  |
| videocamera | uil |  |
| vishengel | veiligheidsbril |  |
| voet | verfblik |  |
| voetbalschoen | vlieger |  |
| vogelnest | voetbal |  |
| vork | vrachtwagen |  |
| waaier | waterflesje |  |
| wasbak | waterput |  |
| wasknijper | wereldbol |  |
| watermeloen | wijn |  |
| wc | wijnglas |  |
| weegschaal | wol |  |
| wereldkaart | wortel |  |
| windmolen | zaag | |
| zoutvaatje | zeilboot | |
